# Perinatal Women’s Perception of Maternal Health Information Quality on Digital Media: Scoping Review

**DOI:** 10.2196/67620

**Published:** 2025-07-02

**Authors:** Bowen Li, Ningning Jin, Yingli Wang, Xiaoni Hou, Jing Meng, Yihong Zhang

**Affiliations:** 1 School of Nursing Beijing University of Chinese Medicine Beijing China; 2 Peking Union Medical College Hospital, Chinese Academy of Medical Sciences & Peking Union Medical College Beijing China

**Keywords:** information quality perception, information service, maternal and infant health, digital media, scoping review

## Abstract

**Background:**

Perinatal women are increasingly turning to digital media for maternal health information; however, concerns regarding the quality of this information persist. Understanding perinatal women’s perceptions of information quality is essential for enhancing the effectiveness of information services.

**Objective:**

This review aims to (1) identify the key features that perinatal women focus on when perceiving the quality of maternal health information on digital media and (2) summarize the quality issues with maternal health information on digital media that perinatal women have reported.

**Methods:**

A scoping review was conducted following the PRISMA-ScR (Preferred Reporting Items for Systematic Reviews and Meta-Analyses Extension for Scoping Reviews) guidelines using PubMed, Web of Science, Embase, Scopus, and ScienceDirect databases (2000-2024). The search strategy combined the following four conceptual clusters using Boolean operators: (1) perinatal population terms (“pregnant women,” “expectant mothers,” and “perinatal”), (2) information-related terms (“information,” “education,” and “resource”), (3) perception-related terms (“perception,” “experience,” and “expectation”), and (4) digital media terms (“online,” “social media,” and “app”). Thematic analysis was used for data synthesis.

**Results:**

From 5290 records identified, 30 (0.57%) articles were selected for inclusion in this review. The perceived quality features of information can be categorized into four distinct aspects: (1) information providers, which encompasses 2 features, transparency and authority; (2) information content, consisting of 9 features, trustworthiness, evidence based, timeliness, comprehensiveness, need-based relevance, practicality, motivational simulation, emotional supportiveness, and cultural sensitivity; (3) information presentation, which includes 3 features, understandability, attractiveness, and conciseness; and (4) information platforms, comprising 3 features, user-friendly navigation, proactive delivery, and interactivity. Furthermore, several perceived quality issues associated with these aspects were noteworthy. Specifically, (1) quality issues regarding information providers primarily pertained to their lack of credibility; (2) quality issues related to information content encompassed an overwhelming volume of information, inaccuracies, lack of scientific evidence, prevalence of contradictory information, insufficient breadth and depth, a mismatch between content and the needs of women, and information that induces negative emotions; (3) presentation issues manifested as difficulties in understanding the information; and (4) quality issues regarding information platforms included poor usability and the commercialization of these platforms.

**Conclusions:**

Our review identifies 17 key quality features across various dimensions that are valued by perinatal women. While there are similarities with quality indicators found in general health information, the unique quality features shaped by the specific characteristics of the perinatal population cannot be overlooked. These distinctive attributes highlight the importance of tailoring maternal health information to meet the unique needs and preferences of perinatal women. Although digital media information services offer many benefits, this study indicates that perinatal women are dissatisfied with the quality of existing maternal health information. Clearly, future efforts should focus on integrating perinatal women’s perceptions of information quality to ensure ongoing improvements in information quality.

## Introduction

### Background

Pregnancy, childbirth, and motherhood are important turning points in a woman’s life cycle. Sudden role transitions, unfamiliar physical and physiological changes, and uncertain pregnancy outcomes often lead to a series of negative emotions for perinatal women [1,2]. Therefore, adequate information is needed to cope with these health issues and provide a sense of security and reassurance during this period [3,4]. With the prevalence and evolution of information technology, more women have turned to digital media for communication, exchange, and reciprocity of maternal health information [5-7]. Digital media refers to media created, stored, and transmitted in a digital format, which facilitates the creation, dissemination, and interaction of information [8]. Leveraging their notable advantages in availability, interactivity, flexibility, and convenience, these digital media platforms have emerged as alternative sources to address the limited direct communication between women and their health care providers [3,9,10]. In addition, when it comes to some private or sensitive topics, perinatal women from culturally and linguistically diverse backgrounds reported preferring to search digital media to obtain more unbiased information [11,12].

According to survey data, 63% to 80.6% of perinatal women based their management and decision-making related to maternal and infant health on information obtained from digital media [13,14]. Therefore, it is crucial to ensure that this information is of high quality. More recently, several studies have assessed the quality of maternal health information on digital media from a professional perspective using universal online health information quality assessment tools, such as DISCERN, the Silberg criteria, and readability formulas. Most results revealed that information on specific maternal themes was unsatisfactory regarding accuracy, readability, reliability, and completeness [15-21]. However, considering that perinatal women are the recipients of maternal health information, a key factor in maximizing the effectiveness of this information is staying attuned to their perceptions of its quality. Therefore, in addition to professional quality assessment, it is essential to deeply explore how perinatal women perceive the quality of maternal health information on digital media.

Multiple studies have shown that perinatal women often perceive the quality of information based on their own subjective criteria rather than discussing it with their health care providers, leading to significant heterogeneity in the results [8,22,23]. A survey by Lagan et al [14] found that 81% of women checked the official accreditation and credentials of information providers while also noting their affiliated institutions. The studies by Whitworth et al [24,25] revealed that women perceived information as high quality when it aligned with their needs and preferences, was easy to understand, and provided practical guidance. In addition, they appreciated the variety of functions provided by digital media platforms, which facilitated their access to maternal health information [26]. In summary, perinatal women focus on the quality of maternal health information from various perspectives. It is noteworthy that while there were overlaps between the indicators in universal assessment tools and the key points of women’s perceived quality of maternal health information, there were also differences.

To date, numerous studies have explored women’s motivation for using digital media during the perinatal period, as well as the diverse maternal health information they have acquired from this medium. However, it is essential to emphasize that women’s perception of the quality of the information they receive is a critical factor that ultimately affects its value [4,11,27]. Therefore, this critical dimension necessitates clarification and attention. Furthermore, to our knowledge, there are no assessment tools that specifically address the quality of maternal health information on digital media, and even fewer tools have been developed based on women’s perspectives. As mentioned earlier, although previous studies have investigated perinatal women’s perceptions of maternal health information quality, a comprehensive synthesis of these findings is still lacking in the literature.

### Objectives

This study aims to achieve two objectives through a scoping review: (1) to identify the key features that perinatal women focus on and prefer when perceiving the quality of maternal health information on digital media and (2) to summarize the quality issues related to maternal health information on digital media that perinatal women have reported. The findings of this review will serve as a reference for relevant professionals to enhance the quality of maternal health information provided through digital media. Furthermore, it will provide a foundation for developing a comprehensive and multidimensional assessment tool specifically designed to evaluate the quality of maternal health information.

## Methods

### Study Design

The design of this scoping review was in line with the Arksey and O’Malley framework [28], which consists of five steps: (1) identification of the questions to be addressed; (2) identification of the relevant literature sources; (3) selection of literature sources to be included in this scoping review; (4) recording key themes emerging from the literature; (5) collation, summary, and reporting of the results. In this review, we do not intend to critically appraise and synthesize results to answer a single and precise question. Rather, we aim to provide an overview of women’s perceived quality of maternal health information on digital media. Therefore, we undertook a scoping review instead of a systematic review. The scoping review process and reporting structure were further guided by the PRISMA-ScR (Preferred Reporting Items for Systematic Reviews and Meta-Analyses Extension for Scoping Reviews) checklist (Multimedia Appendix 1) [29].

### Search Strategy

In July 2024, we conducted a literature search focusing on perinatal women’s perceptions of the quality of maternal health information on digital media. The databases used in this review are as follows: PubMed, Web of Science, Embase, Scopus, and ScienceDirect.

The search was limited to papers published from January 1, 2000, to June 2024. To avoid language errors that may affect the evaluation results, we restricted the language to English. The key search terms used were as follows: (pregnant women* or expectant mothers* or pregnancy* or postpartum* or labor and delivery* or antenatal*or perinatal*)and (information* or education* or resource* or service*) and (perception* or need* or experience* or expectation* or value* or behavior* or preference* or wish*)and (online* or digital* or social media* or app* or mobile phone* or website* or Internet*). In addition, we referred to the references in the retrieved studies to obtain more relevant gray literature, journal articles, and books.

### Study Inclusion and Exclusion Criteria

In our review, information quality perception refers to the subjective experience of information quality by perinatal women during the process of acquiring and using information. Digital media include websites, social media (TikTok [ByteDance], WeChat [Tencent], YouTube [Google], Facebook [Meta], and online forums), search engines, and maternity and infant apps. Maternal health information encompasses all information related to maternal and infant health during the preparatory stage of pregnancy, throughout pregnancy, during childbirth, and within 2 years following delivery.

The inclusion criteria were as follows: (1) studies that involved women who were planning pregnancy, pregnant women, and women within the first 2 years post partum as participants; (2) studies that identified women’s perceptions of the quality of maternal health information on digital media; (3) studies that were published in English; (4) original research studies, including qualitative, quantitative, and mixed methods studies. Studies that were reviews, conference summaries, policy interpretations, and advertisements were excluded.

### Study Selection

The retrieved studies were imported into NoteExpress (version 4.0; Aegean Software), and duplicates were removed. The studies were screened according to the inclusion and exclusion criteria by reviewing the titles and abstracts. After the initial screening, the full texts of the studies that met the criteria were imported, and the full texts were assessed for rescreening. The process was conducted independently by 2 reviewers (BL and YW), and in case of disagreement, a third reviewer (XH) made the final decision.

### Data Extraction and Synthesis

Data were extracted from the studies independently by 2 reviewers and organized using a data extraction form. The following information was collected: (1) author, (2) year of publication, (3) country, (4) ethnicity, (5) target population, (6) method of data collection, (7) sample size, (8) the features of information quality, and (9) the quality issues of information.

In our study, we used the thematic synthesis analysis method articulated by Thomas and Harden [30]. This approach encompasses three key stages: (1) line-by-line coding of the articles to capture relevant components, (2) development of descriptive themes, and (3) formulation of analytical themes. The process unfolded as follows: initially, all pertinent studies were meticulously examined and coded to extract preliminary findings and themes. Subsequently, these findings were categorized into descriptive themes that reflected the core elements and insights articulated within each study. Finally, we conducted a deeper analysis of the descriptive themes to distill analytical themes that encapsulated overarching concepts across the body of research. During this process, we also drew upon the information ecology theory [31], which posits that an information ecosystem consists of 3 fundamental components: information (encompassing both content and presentation), human factors, and environmental factors. On the basis of this, we categorized quality features and quality issues of maternal health information into four dimensions: (1) quality of information providers (including both professional sources such as medical institutions, physicians, nurses, and other healthcare professionals, as well as non-professional sources such as peer mothers, parenting bloggers, and commercial organizations), (2) quality of information content, (3) quality of information presentation, and (4) quality of information platforms. Two reviewers conducted this process independently, and the results were discussed by our research group.

## Results

### Study Characteristics

**Figure 1:**
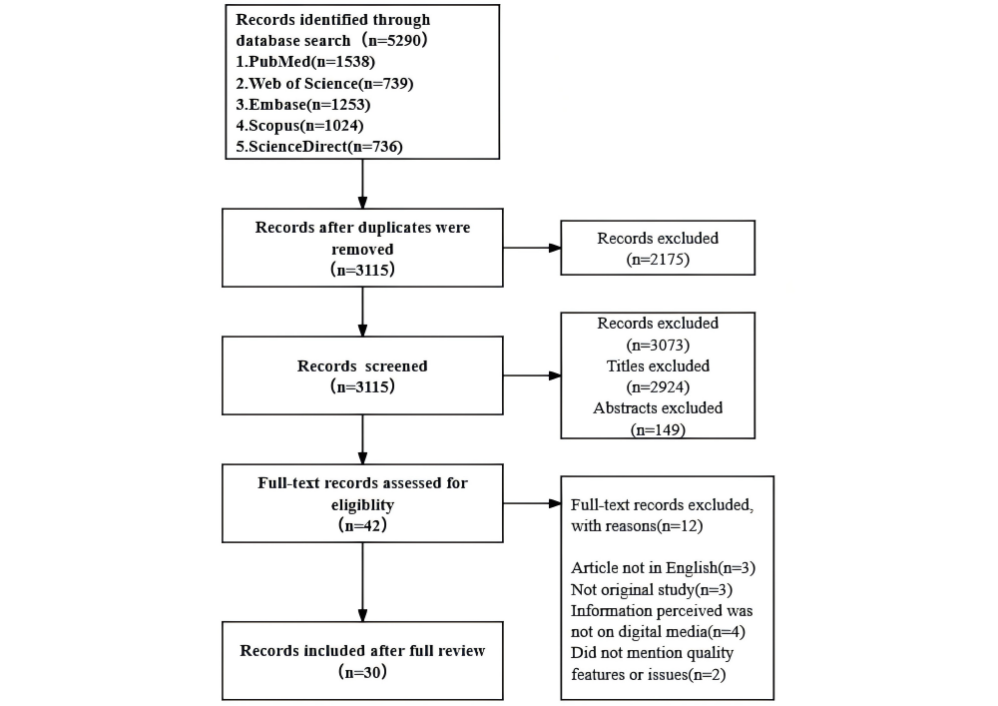
Study flow diagram.

A total of 5290 records were initially retrieved through database searches. After the removal of 2175 (41.12%) duplicates, the titles and abstracts were reviewed against the established inclusion criteria. This process resulted in the identification of 42 (0.79%) articles for full-text screening. Of these, 12 (0.23%) articles were excluded upon review. Eventually, 30 (0.57%) articles were included in this scoping review (Figure 1).

### Characteristics of the Included Studies

The characteristics of the 30 included studies are presented in Table 1. The research designs for the studies included 5 (17%) mixed methods studies, 19 (63%) qualitative studies, and 6 (20%) quantitative studies. Although the literature search began in 2000, only articles published from 2010 onward were included in the final review. This restriction likely reflects the rise of digital media during this period, highlighting a significant evolution in how maternal health information is disseminated and consumed. The distribution of included studies was as follows: Australia (6/30, 20%) [8,25,32-35], the United States (4/30, 13%) [23,36-38], Belgium (2/30, 7%) [11,39], Canada (2/30, 7%) [40,41], China (3/30, 10%) [7,42,43], England (2/30, 7%) [44,45], France (1/30, 3%) [46], Germany (1/30, 3%) [47], Greece (1/30, 3%) [26], Iran (1/30, 3%) [48], South Korea (1/30, 3%) [2], the Netherlands (2/30, 7%) [49,50], Qatar (1/30, 3%) [51], Switzerland (1/30, 3%) [52], Thailand (1/30, 3%) [53], and 1 (3%) study was conducted across 24 countries [14]. Among these, 11 (37%) studies specifically investigated race or ethnicity [14,23,25,37,38,40,41,45,49,50,52].

**Table 1:** Characteristics of the included studies.

Study	Country	Target population	Population type	Study design	Sample size, n
Al-Dahshan et al [51], 2022	Qatar	Pregnant women	—^a^	Quantitative study (questionnaires)	327
Snyder et al [41], 2020	Canada	Pregnant women and postpartum women	Canadian, European, Inuit, Métis, and White	Mixed methods study (questionnaires and interviews)	Questionnaires: 97 and interviews: 10
Arcia et al [23], 2019	United States	Pregnant women	African, American, Black, Hispanic, mixed, and Non-Hispanic White	Qualitative study (focus groups)	16
Bianchi et al [46], 2016	France	Pregnant women	—	Qualitative study (focus groups)	40
Diezi et al [52], 2023	Switzerland	Pregnant women	Non-Switzerland and Switzerland	Qualitative study (interviews)	15
Gourounti et al [26], 2022	Greece	Pregnant women	—	Qualitative study (focus groups)	13
Grenier et al [40], 2021	Canada	Pregnant women	European, mixed and other	Qualitative study (focus group)	66
Hearn et al [33], 2013	Australia	Pregnant and postpartum women	—	Qualitative study (focus groups and interviews)	110
Hearn et al [34], 2014	Australia	Pregnant and postpartum women	—	Qualitative study (interviews and focus groups)	Interviews: 53 and focus groups: 67
Henrich et al [37], 2024	United States	Pregnant and postpartum women	Asian, Black, Hispanic, multiracial or American Indian, and White	Mixed methods study (questionnaires and interviews)	Questionnaires: 257 and interviews: 13
Jacobs et al [49], 2019	Netherlands	Women who are trying to conceive and those who are	Dutch and non-Dutch	Quantitative study (questionnaires)	365
McLeish et al [45], 2020	England	Pregnant women	Asian, Black, mixed, and White	Qualitative study (interviews)	40
Xu et al [43], 2024	China	Pregnant women with gestational diabetes mellitus	—	Mixed methods study (questionnaires and interviews)	Questionnaires: 235 and interviews: 11
Lagan et al [14], 2010	24 countries	Pregnant women	Africa, Asia, Europe, North America, Oceania, and South America	Mixed methods study (questionnaire and focus groups)	Questionnaire: 53,550 and focus groups: 56
Lee and Lee [2], 2022	Korea	Pregnant women	—	Quantitative study (questionnaires)	302
Guerra-Reyes et al [38], 2016	United States	Postpartum women	Asian, Hispanic, mixed, and White	Qualitative study (interviews)	10
Lupton [8], 2016	Australia	Pregnant and postpartum women	—	Qualitative study (focus groups)	36
Maher et al [35], 2018	Australia	Primigravid pregnant women	—	Qualitative study (interviews)	16
McCarthy et al [44], 2020	England	Pregnant women	—	Qualitative study (interviews and focus groups)	31
Ebinghaus et al [47], 2024	Germany	Pregnant women	—	Qualitative study (focus groups)	25
Nuampa et al [53], 2024	Thailand	Postpartum women	—	Qualitative study (focus groups)	48
Slomian et al [11], 2017	Belgium	Postpartum women	—	Qualitative study (interviews and focus groups)	22
Slomian et al [39], 2017	Belgium	Postpartum women	—	Quantitative study (questionnaires)	349
Taheri et al [48], 2021	Iran	Pregnant women	—	Qualitative study (interviews)	18
Vamos et al [36], 2019	United States	Pregnant women	—	Qualitative study (focus group)	17
Vogels-Broeke et al [50], 2022	Netherlands	Pregnant women	Hispanic, Latina, and other	Quantitative study (questionnaires)	1922
Walker et al [32], 2022	Australia	Preconception women	—	Qualitative study (interviews)	12
Wang et al [7], 2019	China	Pregnant women	—	Mixed methods study (questionnaires and interviews)	Questionnaires: 535 and interviews: 28
Whitworth et al [25], 2024	Australia	Pregnant women	Australia and non-Australia	Mixed methods study (questionnaires and interviews)	Questionnaires: 38 and interviews: 5
Zhu et al [42], 2019	China	Pregnant women	—	Qualitative study (interviews)	20

^a^Not available.

The included studies varied significantly in sample sizes, ranging from 5 to 53,550 participants, comprising mixed groups of pregnant and postpartum women. The cohorts consisted of mixed groups of perinatal women. Specifically, 18 (60%) studies focused exclusively on pregnant women [2,7,14,23,25,26,35,36,40,42,44-48,50-52], while 5 (17%) studies centered on postpartum women [11,38,39,43,53]. In addition, 5 (17%) studies included both pregnant and postpartum women [8,33,34,37,41]. The remaining 2 (7%) studies included women who were trying to conceive [32,49]. Among the 30 included studies, 28 (93%) reported on women’s perceived quality features of maternal health information, and 22 (73%) identified existing quality issues as highlighted by the participants.

### Perceived Information Quality Features

#### Overview

The perception of information quality among perinatal women can be categorized into 4 distinct aspects: information providers, information content, information presentation, and information platforms. The specific quality features are illustrated in Table 2.

**Table 2:** Quality features of maternal health information perceived by perinatal women.

Feature			Definition		References		Studies (n=30), n (%)
**Quality features of information providers**							
	Transparency	Information providers disclose their identity, affiliations, and professional certifications in detail and possess official accreditation, ensuring that all elements constituting the provider are credible.		[8,14,26,33-35,37-39,41,43,44,47,51,54]		15 (50)	
		Information providers demonstrate a recognized level of expertise in the field of maternity and are affiliated with reputable, well-known organizations and institutions.		[7,8,14,23,26,33,34,36-38,42,47,49,51,54]		15 (50)	
**Quality features of information content**							
	Trustworthiness	Information exhibits a series of elements that demonstrate its authenticity and reliability.		[2,11,14,23,26,32,34,35,37,41-44,47,49,51,54]		17 (57)	
	Evidence based	Information is supported by evidence from literature reports, experimental data, clinical guidelines, standards, and other scientific materials.		[7,32,34,44,47,51,54]		7 (23)	
	Timeliness	Information is published in close proximity to the present date, with timely updates, and reflects the latest research findings.		[14,25,26,33,37,38,43,49,51,54]		10 (33)	
	Comprehensiveness	Information covers a wide range of topics related to pregnancy, childbirth, and the postpartum period, with necessary, in-depth aspects of each topic addressed.		[7,8,26,32,34,35,37,38,42,49,53]		11 (37)	
	Need-based relevance	Information addresses the universal needs while also considering the individualized needs of diverse characteristics of women.		[7,8,23,32,33,36,37,39,41,44,47,48]		12 (40)	
	Practicality	The information includes practical advice and solutions, along with concrete tools, instruction, and examples that ensure women effectively take relevant actions, address real-world problems, and achieve tangible benefits.		[7,8,14,25,33-37,39,45,50,53]		13 (43)	
	Motivational stimulation	Information includes persuasive elements that effectively motivate women to adopt and implement relevant recommendations.		[11,25,32,33,35,43,53]		7 (23)	
	Emotional supportiveness	Information includes emotionally supportive elements that promote positive emotional experiences when applying it.		[2,8,23,25,35,42-44,52]		9 (30)	
	Cultural sensitivity	Information aligns with the cultural values, beliefs, and practices related to maternity and parenting that are prevalent among the target population of perinatal women.		[26,35,37]		3 (10)	
**Quality features of information presentation**							
	Understandability	Information is presented in a way that incorporates elements facilitating understanding, catering to the diverse health literacy levels of perinatal women.		[25,26,33,35,38,41,43,49,51,52,54]		11 (37)	
	Attractiveness	Information is presented in an engaging and visually appealing manner, using a tone that is approachable and enjoyable.		[8,32,43]		3 (10)	
	Conciseness	Information is presented in a clear, concise, and organized manner, with each piece of content being brief and to the point, in alignment with women’s reading habits and preferences.		[26,33,35]		3 (10)	
**Quality features of information platforms**							
	User-friendly navigation	Platforms provide easy-to-use navigational functions that simplify the process of accessing information, enabling women to quickly locate desired content within the platform as well as relevant external links and attachments.		[25,32,33,35,37,39,41,43]		8 (27)	
	Proactive delivery	Platforms provide the capability to track women’s current stage as well as maternal-child health, proactively delivering relevant and targeted information.		[7,8,23,25,32,33,37,38,43]		9 (30)	
	Interactivity	Platforms provide multiple interactive channels, facilitating communication among women, information providers, local perinatal peers, and other relevant stakeholders.		[7,8,23,25,32-35,37,42-44,49,53]		14 (47)	

#### Quality of Information Providers

##### Overview

Studies indicated that perinatal women primarily obtained maternal health information from digital media, sourced from a diverse range of entities: government agencies; universities; medical institutions; commercial organizations; as well as health care providers, such as obstetricians, pediatricians, and midwives. In addition, informal individuals, including other perinatal women and mother bloggers, were also seen as important information sources [32,35]. Furthermore, our review revealed that women perceived the quality of various information primarily based on its transparency and authority.

##### Transparency

Transparency involves the clear and open disclosure of personal information by the providers, encompassing various elements that constitute an individual’s true identity. In our review, we found that women placed substantial emphasis on official accreditation and the disclosure of information providers’ affiliation, credentials, and expertise [14,26,37-39,41,43,44,51,54]. It is worth noting that women’s opinions regarding commercial information providers varied. Some women actively shunned these information providers because of their commercial nature [8,34,47]. Conversely, a few women perceived them as credible sources because of their high accountability, given their strong motivation to enhance women’s trust and thereby increase product purchases [35].

##### Authority

Perinatal women commonly perceived the authority of information providers as a crucial factor that directly determined the quality of the information content. In particular, women most frequently valued maternal health information provided by well-known professionals and formal organizations over information from unknown sources. They emphasized that authoritative information providers not only enhanced their sense of identity but also assisted in alleviating anxiety regarding health concerns [7,8,14,23,26,33,34,36-38,42,47,49,51,54].

#### Quality of Information Content

##### Overview

Information content quality reflects women’s focus on the features of the information itself and primarily encompasses 9 aspects: trustworthiness, evidence -based, timeliness, comprehensiveness, need-based relevance, practicality, motivational stimulation, emotional supportiveness, cultural sensitivity.

##### Trustworthiness

While women might have inherent limitations in objectively assessing the accuracy of maternal health information, they often perceive the quality of information through the alternative feature of trustworthiness. Our review identified several factors that contributed to women’s perception of trustworthiness: professional reviews [11,37,43,49,54]; consistency with content from other digital media sources [7,11,23,33,35-37,41-43,48,49,54,55]; validation by fellow women [23,44,49]; endorsements by family, friends, and their health care providers [23,32,35,49,54]; and high popularity [2,32,42,51,54] as reflected in metrics such as view counts, downloads, follower numbers, and likes. In addition, women perceived the trustworthiness of information based on their previous knowledge [23,35,49] and intuition [23,49].

##### Evidence Based

Evidence is a crucial reference for establishing the validity of content. While the best evidence typically forms the foundation for medical treatment and care provided by professionals, several studies revealed that perinatal women also viewed evidence as a criterion for perceiving the quality of information on digital media [32,34,44,47]. Across the studies, women demonstrated a strong preference for adopting information that was supported by references [51,54] and endorsed by internationally recognized “gold standards” and guidelines rather than information that relied solely on personal opinions [7].

##### Timeliness

The perception of timeliness among perinatal women primarily hinged on factors such as the date of publication, update frequency, and the incorporation of the latest research findings [25,26,37,38,43]. Several studies highlighted women’s repeated calls for maternal health information that is timely, regularly updated, and reflects cutting-edge insights on maternity and parenting [14,33,49,51,54].

##### Comprehensiveness

Perinatal women perceived the comprehensiveness of maternal health information based on its dual features of breadth and depth. Specifically, information should encompass various topics throughout the entire process of pregnancy, childbirth, and postpartum [26,32,35,37,38,42,49]. Moreover, the extent of thoroughness in the information presented within each topic was highly valued [7,8,34,35,37,53]. Women expressed that diverse and comprehensive information empowered them to make informed decisions, engage in self-management, and navigate this enduring and intricate period with confidence, which is therefore recognized as high-quality information.

##### Need-Based Relevance

Several studies showed that women highly valued maternal health information that caters to their universal information needs, such as diet and nutrition, physical exercise, fetal development, and labor pain relief [7,32,33,39,41]. In addition, women appreciated information that was oriented toward their personalized needs based on their health status, lifestyle habits, and other personal preferences. This feature is particularly emphasized by specific groups of women, including those with maternal-fetal health conditions, those following specialized diets (eg, vegetarian, pescatarian, flexitarian, or gluten free), and immigrant populations [8,23,32,36,37,44,47,48].

##### Practicality

Perinatal women described practicality as the practical value of maternal health information, emphasizing its ability to provide valuable content that not only informs decision-making but also facilitates the adoption of health behaviors following the acquisition of information [14,34-36,39,50,53]. They generally valued information that included practical tools, step-by-step guidance, and demonstrations [8,33,37,45]. Furthermore, our review found that when confronted with various real-life issues, women appeared to appreciate the information shared by others who had similar experiences rather than relying solely on professional information [7,8,25,35].

##### Motivational Stimulation

Studies have indicated that not all maternal health information encountered by women on digital media is embraced and internalized as personal beliefs and skills. Information that features motivational stimulation serves as a crucial factor, influencing the use intention and effect [11,25,32,33,35,53]. Specifically, women desired not only guidance on what to do or not but also required detailed explanations, including the reason, benefits, risks, and drawbacks, which promoted the persuasiveness of information. Moreover, the inclusion of cases involving fellow women in similar conditions, regardless of their successful or failed outcomes, could enhance the inspiring and cautionary aspects of the information, thereby increasing women’s compliance with the provided content [43].

##### Emotional Supportiveness

Our review found that receiving emotional support is a key reason why women seek maternal health information on digital media [2]. Consequently, information that provides support in an equitable and compassionate manner, offers words of solace and empathy, and shows respect for women’s present circumstances is perceived as high quality [8,24,35,43]. Conversely, information that adopts a didactic “should-do” tone and potentially increased fear and anxiety is viewed as having a “nocebo effect” by women and is likely to be disregarded outright [8,23,42,44,52].

##### Cultural Sensitivity

Cultural sensitivity reflects how well maternal health information resonates with the cultural backgrounds of different groups of women. Several studies have demonstrated that the diverse customs and philosophies surrounding maternity and parenting are deeply embedded in cultural differences, which in turn affect women’s perceived quality of the information. In general, information that respects and acknowledges these cultural variations is more likely to be adopted and favorably appraised by women [26,35,37].

#### Quality of Information Presentation

##### Overview

Maternal health information available on digital media possesses both intrinsic professional attributes and extrinsic presentation attributes. Our review found that women perceived the quality of information presentation based on 3 key features: understandability, attractiveness, and conciseness.

##### Understandability

The understandability of maternal health information is critical, as it ensures that perinatal women, regardless of their health literacy levels, can easily comprehend, interpret, and effectively use information obtained through digital media [26,38,41]. Our review found that women prioritized the use of plain and clear language to simplify complex concepts and reduce the time needed to comprehend the information [33,51]. In parallel, a clear organization and layout facilitated easier navigation, which was highly appraised by women [25,54]. In addition, women also valued the inclusion of visual aids, such as images, graphs, videos, and short film clips, which enhanced content retention and rendered complex information more accessible [25,35,43,49,52].

##### Attractiveness

Perinatal women perceived the attractiveness of information mainly based on 2 aspects: the overall esthetic appeal of the information presentation and a recreational rather than lecture-style approach [8,32,43]. For those women who peruse maternal health information on digital media as a leisure activity, it is essential that engaging elements are present to make them feel engaged and delighted. These elements can prompt them to click or explore further related content.

##### Conciseness

Our review found that while perinatal women desired to obtain sufficient maternal health information, they emphasized that simple and concise presentation was also an important criterion for their perception of information quality. Given their reliance on digital media during fragmented time slots, lengthy and convoluted information was not conducive to better assimilation of the content. In contrast, women were more inclined to choose information that was organized into concise sections, each addressing a specific issue or delivering a single point of knowledge [26,33,35].

#### Quality of Information Platforms

##### Overview

The digital media platforms used by women to obtain maternal health information include maternity and infant apps [32], websites [25,32,35,36,41], social media (such as YouTube, Facebook, Instagram and WeChat) [2,23,25,26,32,35,36,43,47,53,55], and search engines (such as Google and Baidu) [2,8,23,25,32,35,36,41,43,55]. Women primarily perceived the quality of these platforms based on 3 features: user-friendly navigation, proactive delivery, and interactivity.

##### User-Friendly Navigation

User-friendly navigation significantly enhanced the ease and speed with which perinatal women could navigate various areas on digital media platforms. Consequently, this feature was regarded as a hallmark of “high-quality” platforms by women [32,35,37,39,41,43]. In addition to swiftly accessing the required content within the platform through specific functions, such as search bars and categorized tags, women expressed a strong desire for platforms that incorporated external links and attachments, enabling easy navigation to additional content via a simple click or download [24,33-35].

##### Proactive Delivery

It was acknowledged that women often struggled to accurately grasp what they would encounter and how to cope with it during each stage of the perinatal period. As a result, women highly valued the function of digital media platforms that proactively provide staged maternal health information, which allows them to understand their current situation without having to wait for a need or crisis to arise [7,25,32,33,38,43]. Furthermore, women indicated that platforms could track maternal and child health data and proactively deliver tailored information that better meets their expectations for high-quality services, as compared to requiring them to seek information actively [8,23,37,43].

##### Interactivity

Studies have shown that women value the interactive features of platforms that allow engagement with a diverse group of individuals [23,33,49]. Women highlighted that specific areas on these platforms, such as comment sections or message boards, enhanced their ability to seek additional support from information providers, particularly professionals, for further questions after accessing maternal health information [8,43,44,53,56]. They also expressed enthusiasm for sharing experiences and exchanging information with other women using these platforms [7,8,25,32,35,42,44]. Furthermore, strong interactivity was also enhanced by the locally focused nature of platforms, which facilitated both online and offline connections with others who receive assistance from the same health care providers or reside in the same community [8,23,37,43].

Regarding the immediacy of interactivity, women’s perspectives varied. Some preferred timely feedback, considering it as a crucial determinant of quality perception [34,37,42,43]. However, others did not prioritize the speed of response, instead viewing the act of posing questions as a way to alleviate stress and seek reassurance [44].

### Perceived Information Quality Issues

#### Overview

Our review found that existing maternal health information on digital media did not fully meet the expectations of perinatal women regarding quality, and they were attentive to numerous quality issues when accessing, understanding, and using information.

#### Quality Issues of Information Providers

Women expressed skepticism regarding the credibility of information providers on digital media platforms, particularly because of the lack of identity disclosures [8,40,46]. Some even categorically deemed certain sources as unreliable [11,41,50]. Furthermore, statements such as “that may be literally just someone random wrote it” reflect a prevalent sentiment among women who question the trustworthiness of information content, primarily because of the anonymous or nonexpert nature of these information providers [7,41,43].

#### Quality Issues of Information Content

##### Overwhelming Amount of Information

Women expressed a significant concern regarding the overwhelming amount of maternal health information available on digital media. They found that valuable information was interspersed with irrelevant and repetitive content, which left them feeling overburdened and inundated [32,36,38,43,44]. Moreover, women described navigating through this vast quantity of information as inefficient and time-consuming, frequently noting the lack of guidance for swiftly locating high-quality content [3,47].

##### Inaccuracy of Information

Several studies indicated that women’s trust in maternal health information was eroded because they often found that not all content was accurate. Women reported encountering erroneous and misleading information, which increased their confusion [7,14,32,35,39,44,48]. Consequently, because of concerns about being misled by such information, women expressed a preference for consulting health care providers directly regarding serious maternal and infant health issues rather than extensively searching on the digital media [26].

##### Lack of Scientific Evidence for Information

Women expressed dissatisfaction with information that lacked sufficient scientific evidence [35], perceiving such content as potentially biased and invalid. This concern was particularly pronounced regarding information shared by experienced women, leading to more cautious selection because of its unsupported nature [7]. Moreover, women reported that the lack of scientific rigor was particularly concerning on websites and maternity and infant apps, adversely affecting their intention to use these sources for health-related decisions [38,48].

##### Prevalence of Contradictory Information

Women identified the prevalence of contradictory information on digital media as a critical quality issue, which compounded their feelings of uncertainty [42-44,46,47]. This inconsistency was particularly evident when comparing guidance from health care providers to the recommendations available on digital media [34]. Furthermore, even within a single information source, women reported that there was contradictory content [7]. Consequently, this complexity in information hindered their decision-making processes and led to heightened frustration and anxiety.

##### Insufficient Breadth and Depth of Information

Studies revealed that women identified significant limitations in the quality of maternal health information regarding the depth and breadth of content. While the information obtained from digital media offered certain advantages, women reported that the abundance of brief, generic, and ambiguous content was not conducive to knowledge acquisition and the alleviation of negative emotions [35,43,51]. In addition, women frequently found that information on digital media was incomplete and fragmented, which consequently hindered their access to continuous information support throughout the whole perinatal period and made it difficult to locate a single comprehensive source for all information on specific topics [38,39,41,45].

##### Mismatch Between Information Content and Women’s Needs

Women reported significant discrepancies between the maternal health information available on digital media and their nuanced and individualized needs [35,41,42,44]. It has been emphasized that women generally have distinct information needs at different stages of their perinatal period. Moreover, even within commonly discussed topics, information needs remain highly personalized [48]. However, many information providers failed to adequately address the specific and detailed needs of women, resulting in information that lacked relevance. Consequently, women struggled to access effective support for their unique concerns and therefore perceived such information as poor quality [38].

##### Information That Triggers Negative Emotions

While women desperately needed fact-based maternal health information, they criticized the scaremongering approach often adopted, with negative and horror stories dominating the communication of information [35]. They further reported increased anxiety and worry after engaging with such information on digital media, especially on online discussion forums and maternity and infant apps, where they were abruptly confronted with distressing content, such as stories of miscarriages or fetal growth restriction [7,43,51].

#### Quality Issues of Information Presentation

Although maternal health information is highly specialized and valuable, it has gone unrecognized among perinatal women because of the lack of elements that ensure the content is easy to understand. Women reported that information presented in overly complex language, poorly organized, and filled with jargon, which some describe as “doctor-ish,” often reduced their patience and interest in engaging with the content [23,40].

#### Quality Issues of Information Platforms

##### Poor Usability of Platforms

Women perceived that quality-assured digital media platforms were not always user-friendly, making it difficult to quickly locate relevant maternal health information when required [25,33,40]. Some applications were criticized for being overly complex, requiring steps such as registration, precise keyword input, and consuming substantial storage space on mobile devices [43]. In addition, many medical websites often lacked mobile compatibility and were affected by loading issues, which limited the benefits women could gain from valuable information [41].

##### Commercialization of Platform

Perinatal women criticized the increasing commercialization of digital media platforms, characterized by an abundance of advertisements and numerous paid content options. Considering the financial expenses involved, many women expressed their reluctance to pay for such content, often opting for free resources instead. This preference may result in limited access to the valuable information they require [7,37,38,47,53].

## Discussion

### Principal Findings

In this review, 17 quality features that perinatal women focus on when perceiving maternal health information on digital media have been identified. These features are categorized into 4 distinct dimensions: quality of information providers (2 features), quality of information content (9 features), quality of information presentation (3 features), and quality of information platforms (3 features). Within these categories, 10 perceived quality issues have been identified.

#### Perinatal Women’s Perception of Information Providers: The Crucial Role of Transparency and Authority

The widespread use of digital media has fostered an ecosystem where diverse user groups actively engage in the creation and dissemination of information. This phenomenon exerts a dual effect on women’s perception of information quality. On the one hand, women tend to place a higher value on exposure to diverse voices, fostering a sense of engagement and social support. However, the vast number of information providers on these platforms has resulted in an overwhelming volume of maternal health information for women, making it challenging to identify high-quality content efficiently. This observation aligns with the findings of Soroya et al [57], who demonstrated that information overload imposes a significant cognitive load, which may subsequently trigger negative emotional states and informational avoidance behaviors. Our review indicated that, to address this issue, women commonly emphasized the transparency and authority of information providers, which is also considered important criteria by widely used health information quality assessment tools, such as the Health on the Net code [58], Silberg criteria [59], and Quality Evaluation Scoring Tool [60]. While these quality features are highly valued by both women and experts, women still encountered information providers on digital media who do not disclose their personal background information. Moreover, while women perceive authoritative providers as more credible, the proliferation of nonexpert and unofficial providers on digital media has eroded their trust in maternal health information. Regarding commercial information providers, women exhibit mixed perceptions. The profit-driven nature inherent in commercial entities inevitably casts a shadow of potential bias over the information they provide. Previous studies have documented that maternal health information from commercial information providers generally has lower quality compared to that from official sources [19,20]. However, in recent years, large-scale commercial organizations, such as Dingxiangyuan (a leading Chinese health care institution), and multinational pharmaceutical corporations have rapidly expanded their health information services through digital media platforms. To maintain their reputation and expand their influence, these organizations have increasingly adopted more rigorous content moderation mechanisms. This shift may explain why some women in our review perceived certain commercial information providers as credible sources.

These findings carry implications for multiple stakeholders. First, health care professionals should actively participate in information services on digital media, ensuring their credentials and expertise are explicitly highlighted when creating content. In addition, as commercial information providers, they should uphold high-quality content production standards while consciously maintaining clear boundaries between information services and promotional activities. To enhance credibility, it is recommended that disclosure statements, such as “this content is intended for educational purposes only and does not endorse any commercial products or services,” be explicitly declared by commercial information providers. Digital media platforms should strategically leverage their capabilities to organize professional reviews and prioritize the visibility of high-quality content. Specifically, drawing on the practice of the University of British Columbia Health Care Management Center Portal, platforms could prominently rank professionally crafted and vetted information in easily accessible locations [61]. Meanwhile, to mitigate information redundancy, efforts should be made to establish incentive mechanisms and strengthen intellectual property protections, encouraging the dissemination of original content rather than repetitive or excessively reposted content.

#### Perinatal Women’s Perception of Information Content: Characterized by Group Specificity and Diversity

The quality of maternal health information content is intricately linked to the well-being of both women and their offspring, making it a crucial dimension for women to perceive the quality of information [62,63]. Our review indicated that the “trustworthiness equals accuracy” perception rule is often adopted by women, who have devised various strategies to help them determine which information is trustworthy [14,64]. However, through these strategies, women reported discovering a substantial amount of incorrect, inconsistent, and even conflicting information available on digital media. In this regard, professionals have raised concerns that women may overestimate their ability to judge the trustworthiness of information, often viewing themselves—perhaps inaccurately—as “expert users” capable of selecting and recommending valuable information to others [14,22,65,66]. Therefore, as several researchers have highlighted, health care providers who have close contact with women during the perinatal period should take a leading role in actively recommending, screening, and producing high-quality maternal health information for women [26,67].

In addition, we found that evidence-based support helps women differentiate between accurate and misleading information, as they view evidence as an indicator of academic rigor and validity. Unfortunately, many women struggle with confusion because of the lack of evidence-based information available on digital media, which is frequently perceived by women as low quality. Notably, women often make such judgments solely in the presence of evidence without critically evaluating the currency, level, and rigor of the underlying evidence. In fact, multiple studies have demonstrated that the existing maternal health information on digital media often fails to align with the latest high-level guidelines [15,68]. To effectively address these deficiencies, information providers must take responsibility for ensuring the scientific validity of information from the outset. Guided by the evidence-based strategy, they should systematically and comprehensively retrieve, select, and apply higher-quality evidence. In addition, tools, such as literature trackers, are fully used to monitor the latest research findings and guidelines in the field of maternity, ensuring that the information is updated in a timely manner. Furthermore, digital media platforms should establish robust content moderation mechanisms and provide dedicated feedback channels to effectively identify and filter out outdated and pseudoscientific content [69]. To enhance compliance, platforms should impose stricter penalties that increase the cost of disseminating misinformation. Measures such as account suspensions, financial fines, and legal consequences would collectively cultivate a healthier information ecosystem [70].

It is widely acknowledged that providing complete information for perinatal women can empower them with necessary knowledge, thereby enhancing their self-management and decision-making abilities regarding their health conditions [42]. The perinatal period, characterized by its prolonged duration and numerous complex factors, often presents women with a variety of unpredictable situations. Unlike other patients, perinatal women spend most of their time in nonhospital environments. Consequently, they have unique quality requirements for maternal health information. Specifically, information must be capable of delving into the complex realities they face and aligning with both their general and personalized needs that arise in specific contexts, such as self-management at home, in the workplace, and during travel. However, our review indicates that women often perceive information on digital media as incomplete and fragmented, compelling them to engage in extensive efforts to collect information from multiple sources to bridge existing knowledge gaps [52,53]. In addition, women expressed dissatisfaction regarding the disparity between the supply and demand for maternal health information available on digital media. Previous studies suggested that this dissatisfaction primarily arises from information providers, particularly health care professionals, who tend to prioritize clinical outcome–related information [71-73] while overlooking the nuanced needs of women for a positive and high-quality reproductive experience [42]. These findings underscore that it is imperative to integrate expert perspectives with women’s perceptions to redefine the breadth and depth of maternal health information services. Information providers, especially health care providers, should leverage their frequent clinical interactions with women to explore their information needs systematically. Concurrently, we suggest that researchers leverage question and answer data from digital media platforms and use methodologies such as social network analysis to identify the diverse needs and specific concerns of women with varying characteristics.

Moreover, addressing information needs constitutes only 1 aspect of the problem-solving process. The practicality of information, defined by its real-world applicability, represents a more significant dimension of information acquisition and serves as a critical determinant of its quality [74]. During the perinatal period, women are required to actively engage in various practices, including physical exercise, exerting effort during labor, breastfeeding, newborn care, and handling unforeseen circumstances independently. Therefore, women emphasize the significance of maternal health information that can be practically applied, allowing them to translate theoretical knowledge into practical skills for conducting self-assessments and executing necessary actions. Moreover, in the real-life context of the perinatal period, minor issues may be amplified, prompting women to increasingly seek practical information that directly addresses core issues and yields tangible benefits. The insights derived from this quality feature highlight the necessity to provide content that is not only theoretically robust but also grounded in women’s lived experiences and practically feasible. Furthermore, by leveraging digital media, the integration of technology into content creation can provide information that includes personalized assessment tools, simulation training, and visual guidance, significantly enhancing women’s ability to acquire practical skills and promote essential behaviors.

In addition to practicality, individual motivation is also an important facilitator for behavioral change following information access [75]. A study conducted by Nuampa et al [53] indicated that women require motivation to enhance their satisfaction and engagement when using maternal health information available on digital media. When women understand the implications of following or disregarding the information, they are more likely to engage actively in accepting the information and participating in self-management. This suggests that motivationally stimulating information could effectively foster the continuous improvement of health information literacy among perinatal women. Despite being widely emphasized by perinatal women and domain experts [32,33], the feature of motivational stimulation remains inadequately represented in existing information quality assessment tools. This gap underscores the need for researchers to prioritize its inclusion as a key evaluation criterion within these tools. To enhance the motivational stimulation of maternal health information, information providers should optimize the narrative strategy by providing in-depth explanations that help women better understand the mechanisms, benefits, and potential risks. Such an approach can be enhanced by the incorporation of diverse real-life examples, which encompass both successful cases and cautionary narratives, serving as an effective measure to strengthen intrinsic efficacy. Concurrently, the implementation of gamified reward mechanisms through digital media, such as points, badges, leaderboards, currency, and honorary titles, can effectively augment external motivation by improving the sense of engagement and immersion [76]. In addition, information should be designed to establish clear and achievable goals that promote maternal and infant health outcomes. Digital technologies that facilitate check-in reminders and goal completion logging may also reinforce women’s sense of purpose and self-efficacy, thereby sustaining health-related motivation.

Research indicates that significant emotional fluctuations in women during the perinatal period make emotionally supportive information essential [77]. Our review confirms this, as women reported that information with encouragement, comfort, and humanistic concerns helped them feel relaxed, respected, and understood, positively influencing their perception of information quality. Moreover, information that respects women’s autonomy and offers suggestions in an egalitarian manner, as opposed to prescriptively directing them, was associated with higher satisfaction. Nevertheless, many women criticized current maternal health information on digital media for featuring alarming perinatal “horror stories” and exaggerating low-probability events, which unnecessarily amplified their anxiety and fear. Despite the prevalence of this issue, few studies have systematically evaluated and adequately addressed this gap. Given the significance of this quality feature for perinatal women, we suggest that information providers incorporate elements of empathy into the creation of perinatal health information and optimize narrative techniques that evoke a sense of equality and acceptance. On digital media platforms, sentiment analysis technologies can be effectively used to understand women’s emotional experiences after information acquisition, thereby guiding the optimization of the emotional supportiveness of content design [78]. Existing studies have demonstrated the positive role of big data and artificial intelligence in content moderation. By leveraging these technologies, digital media platforms can effectively identify and filter out exaggerated and fear-inducing content [79]. Furthermore, these platforms can empower perinatal women users by enhancing supervision and reporting mechanisms. When women identify disturbing content, they can effectively report it through dedicated channels.

In our review, we also identified a novel aspect that is often overlooked in the discourse on health information quality: the feature of cultural sensitivity. Several studies indicated that maternal health information, such as optimal delivery methods, dietary preferences and restrictions, and lifestyle management, is significantly influenced by varying values and beliefs across different regions and religious backgrounds [26,35]. For example, Asian women continue to adhere to traditional practices during the postpartum period, particularly "zuo yue zi" (postpartum confinement: a practice where women stay at home and follow specific dietary, lifestyle, and behavioral customs for 42 days or longer after childbirth to aid their physical recovery) a cultural custom that has been preserved through successive generations, whereas such practices are rarely observed among women in Western countries [80]. In addition, in some Latinx communities, many women are more accustomed to using Spanish, highlighting that information content can only be understood and applied when it aligns with their local linguistic style. Consequently, ignoring cultural differences and adopting a “one-size-fits-all” approach can undermine the effectiveness of information for women from specific cultural backgrounds and may lead to perceived cultural conflicts. The study by Chang et al [81] demonstrates that the Chinese immigrants in British Columbia faced barriers to implementing Chinese traditional practices, primarily because of the inadequacy of culturally sensitive information services in local health care systems. Therefore, we suggest that information providers should pay full attention to cultural backgrounds, making them a crucial reference for developing diverse information content. Specifically, they should form content creation teams with multicultural backgrounds and categorize information by cultural groups to help women find resources that match their own cultural contexts. Platforms should also develop multilingual switching functions and provide cultural customization features so that after women register their cultural affiliations, the platform can prioritize delivering information tailored to their cultural backgrounds. In addition, digital media platforms should establish cultural exchange communities. In such communities, perinatal women from diverse backgrounds can share their experiences and cultural practices, which serves as a vital way to understand and enhance the cultural sensitivity of maternal health information.

#### Perinatal Women’s Perception of Information Presentation: The Vital Importance of Elements Emphasizing Clarity, Engagement, and Conciseness

As many women have noted, access to a vast amount of professional maternal health information does not necessarily equate to its comprehension and assimilation. How the content is presented plays a crucial role. Enhancing content understandability through presentation adaptation lowers the health information literacy threshold required for processing maternal health information, enabling women across varying literacy levels to effectively apply the information. Our review indicates that women have a higher evaluation of understandable information that strategically uses accessible language, optimizes layout design, and integrates a diverse array of visual elements. In addition, the fast pace of modern life and the increasing prevalence of fragmentary reading habits have drawn women’s attention toward the conciseness of information presentation. Women noted that content that is overly complex or densely packed can increase cognitive load, limiting their patience and capacity to read, comprehend, and retain all necessary information. This finding aligns with research by Chen et al [82], which emphasizes that the length of health information is closely related to users’ lower willingness to engage and participate. In addition, with the widespread penetration and application of digital technologies, perinatal women are increasingly exposed to content rich in novelty and amusement, which enhances their expectations of information presented in vivid and engaging formats. In particular, in an era characterized by information overload, our review found that attractive information can evoke strong sensory responses from women, not only encouraging deep engagement but also increasing the likelihood of sustained attention and proactive sharing behavior. Previous studies have corroborated the importance of attractiveness. These findings suggest that, in the context of digital media, the external presentation attributes of maternal health information should be adequately considered. This approach addresses the digital divide between the overwhelming volume of information and women’s limited understanding, thereby promoting the widespread dissemination of valuable information in a clear, concise, and engaging manner.

#### Perinatal Women’s Perception of Information Platforms: Highlighting Effective Acquisition, Dissemination, and Interaction of Information

Digital media platforms, as primary mediums for information search and acquisition, significantly influence women’s perceptions of maternal health information. Women highlighted the need for platforms to have strong technical support and device compatibility to quickly access desired information, reducing the time and effort involved in searching for information. This aligns with the study by Zhang and Kim [83], which identified navigational functionality as a crucial factor in user satisfaction with online health information. However, women still faced challenges in conveniently obtaining information because of complicated processes, incompatible devices, and slow response times, which diminished their patience. These findings provide important areas for optimization for digital media platforms regarding navigation and usability. Digital media platforms break down traditional one-way communication barriers, empowering women to engage more actively in information exchange. This participatory shift consequently enhances their perception of information quality. Owing to constraints such as time, transportation, and confidence in face-to-face interactions, women often refrain from seeking assistance from health care institutions for every concern [53]. Instead, they prefer to ask questions and seek information from professionals via digital media. In addition, unfamiliar physical changes, along with feelings of uncertainty and isolation, increase women’s desire to learn from the experiences of others online, particularly those from similar backgrounds within their community who access the same health care services [11]. Moreover, with the support of digital technologies, the information services offered by platforms have evolved from qualitative insights to precise quantitative data. Previous studies have shown that women at different stages of the perinatal period and with varying health conditions prioritize different types of information [2,84]. When women perceive that the information provided does not align with their personal situation, their interest diminishes and their perception of the information quality also declines. These findings suggest that maternal health information included on digital media platforms should be not only continuous and comprehensive but also customizable and proactively delivered based on each woman’s stage and unique circumstances. This can be achieved by collaborating with health care professionals, using feedback mechanisms, and leveraging data analytics.

### Limitations

This review has several limitations that should be acknowledged. First, our literature search was narrowly focused on perinatal women’s perceptions of the quality of maternal health information on digital media, which resulted in a limited number of relevant studies. To mitigate this limitation, we expanded our search criteria to include articles that examined perinatal women’s information-seeking behaviors, experiences, and information needs. During the review process, we carefully screened these full texts to extract relevant content specifically related to the quality features and issues of maternal health information on digital media, as reported by women. Second, some of the studies included mixed participants beyond perinatal women, such as their partners and health care providers, which may introduce bias into perinatal women’s reported perceptions. While we manually extracted women-specific data, the original studies’ aggregated reporting of findings and methodologies that lacked role differentiation (eg, group interviews) may inadvertently cause confusion. To strengthen validity, future reviews should specify inclusion criteria requiring participant stratification at the study design stage and prioritize analysis of datasets with explicitly segregated perspectives. Third, the studies included in this review primarily focused on reporting specific quality features and issues in the literature. However, very few studies have analyzed how information quality priorities may vary among women with different characteristics. In addition, these studies failed to delineate how women’s characteristics mechanistically influence their perceptions of information quality. Future research should further explore the concerns of women from different backgrounds regarding different quality features and issues. This can be achieved through methods such as interview-based qualitative research, latent class analysis, and structural equation modeling constructed based on survey data, thereby providing more targeted measures to improve the quality of maternal health information on digital media.

### Conclusions

This scoping review examined perinatal women’s perceptions of the quality of maternal health information available on digital media. A total of 17 quality features across multiple dimensions—including information providers, information content, information presentation, and information platforms—were identified. By summarizing and elucidating these quality features, this review enhances the understanding of high-quality maternal health information as a distinct category within the broad field of health information. Perinatal women’s quality perceptions encompass established indicators, such as credibility, understandability, and navigability, which are commonly recognized in the evaluation of general health information on digital media. However, the unique characteristics of the perinatal population, such as the extended duration of the perinatal period, significant emotional fluctuations, and considerable cultural variations in childbirth beliefs and practices, impart distinct and pronounced quality features to maternal health information. Therefore, future research should prioritize the integration of health care professionals’ insights with the quality expectations of perinatal women. This collaborative approach is crucial for the development of higher-quality maternal health information, ultimately enhancing health literacy among perinatal women and ensuring the long-term well-being of mothers and their children. In addition, this review identifies 10 prevalent quality issues within maternal health information as reported by perinatal women. The existence of these quality concerns underscores a significant gap in information quality assessment and management. Consequently, it is essential to consolidate women’s perceptions of information quality and to develop comprehensive evaluation tools specifically designed for maternal health information, thereby facilitating the continuous improvement of information quality.

## Appendix

Multimedia Appendix 1 PRISMA-ScR (Preferred Reporting Items for Systematic Reviews and Meta-Analyses Extension for Scoping Reviews) checklist.

## Abbreviations

PRISMA-ScR: Preferred Reporting Items for Systematic Reviews and Meta-Analyses Extension for Scoping Reviews
